# Why it should be "Alzheimer disease" rather than "Alzheimer's disease"

**DOI:** 10.17879/freeneuropathology-2026-9132

**Published:** 2026-03-23

**Authors:** Cinthya Aguero, C. Zachary Klein, Georg Haase

**Affiliations:** 1 MassGeneral Institute for Neurodegenerative Disease, Charlestown, MA, USA; 2 Department of Neurology, Massachusetts General Hospital, Boston, MA, USA; 3 Harvard Medical School, Boston, MA, USA; 4 Institute of Systems Neuroscience, INSERM - Aix-Marseille University, UMS 1106, Marseille, France

**Keywords:** Alzheimer disease, Alzheimer's disease, Down syndrome, Tourette syndrome, Possessive eponym, Non-possessive eponym, ICD-11, AMA style guide, NIH editorial style guide, Exact phrase, All fields, MeSH

## Abstract

The terms "Alzheimer's disease" and "Alzheimer disease" are often used interchangeably in the biomedical literature. Yet this seemingly minor grammatical difference carries implications that extend beyond style: the possessive form, marked by the 's eponym, may imply ownership of a disease by an individual, a notion discouraged by several authoritative medical style guides and international health organizations [1]. In this article, we examine the historical emergence of the term "Alzheimer's disease", analyze the trajectories of the possessive and non-possessive eponyms in PubMed-indexed article titles from 1950 to 2025, and assess how the choice of terminology influences literature retrieval. Our analysis indicates that the possessive form has overwhelmingly dominated the literature for decades. However, searches using "Alzheimer's disease" or "Alzheimer disease" retrieve non-identical, only partially overlapping sets of records in PubMed. We argue that adopting the non-possessive form "Alzheimer disease" would improve conceptual clarity, terminological consistency, and the completeness of literature retrieval, particularly in systematic reviews and meta-analyses.

## Introduction

Alzheimer disease is named after Dr. Alois Alzheimer, a German psychiatrist and neuro-pathologist who, in 1907, made the first detailed description of the condition now commonly associated with progressive memory loss and cognitive decline [3]. Later, he provided a more extensive pathological analysis [5]. His work began with a patient named Auguste Deter, whose unusual symptoms and brain pathology were carefully documented by Dr. Alzheimer. It is therefore natural for many people to think of the disease as "belonging" to Alzheimer, leading to widespread use of the possessive form "Alzheimer's disease." Interestingly, the now widely used abbreviation AD for Alzheimer disease is the same as the initials of Alzheimer's first patient, Auguste Deter (AD), a coincidence that links the modern shorthand designation of the disease to the very first case described.

## Historical aspects

Historically, the Alzheimer eponym was introduced in 1910 by Emil Kraepelin, who coined the term *Alzheimersche Krankheit* in the eighth edition of his psychiatry textbook *Psychiatrie* [5]. Notably, the original German term *Alzheimersche Krankheit* is considered adjectival rather than possessive. The possessive expression "Alzheimer's disease" appeared shortly thereafter in the English medical literature, for example in a 1912 report by Robert Muir Stewart [6], and was subsequently widely adopted in Anglo-American usage.

## Style guidelines and international recommendations

In modern medical writing, style guides and authoritative bodies in healthcare have long discouraged the use of possessive eponyms. The American Medical Association Manual of Style, a gold standard for medical writing, explicitly recommends dropping the possessives in eponyms because a disease is not something that belongs to the person whose name it bears [1]. Instead, the non-possessive form emphasizes that the name honors the individual's contribution without implying ownership.

The World Health Organization reinforces this approach through its International Classification of Diseases, 11th Revision (ICD-11) [2]. The ICD-11 officially uses "Alzheimer disease" as its preferred term, promoting consistency and clarity worldwide. In fact, this movement toward dropping possessives is not new; as early as 1975, The Lancet stated that "the possessive use of an eponym should be discontinued" to encourage a more systematic and standardized approach to medical terminology [7]. Yet, there is no complete consensus among biomedical authorities. The NIH Editorial Style Guide, for instance, continues to recommend the possessive form "Alzheimer's disease" [8].

## Trajectories of the terms "Alzheimer's disease" and "Alzheimer disease"

**Figure 1:**
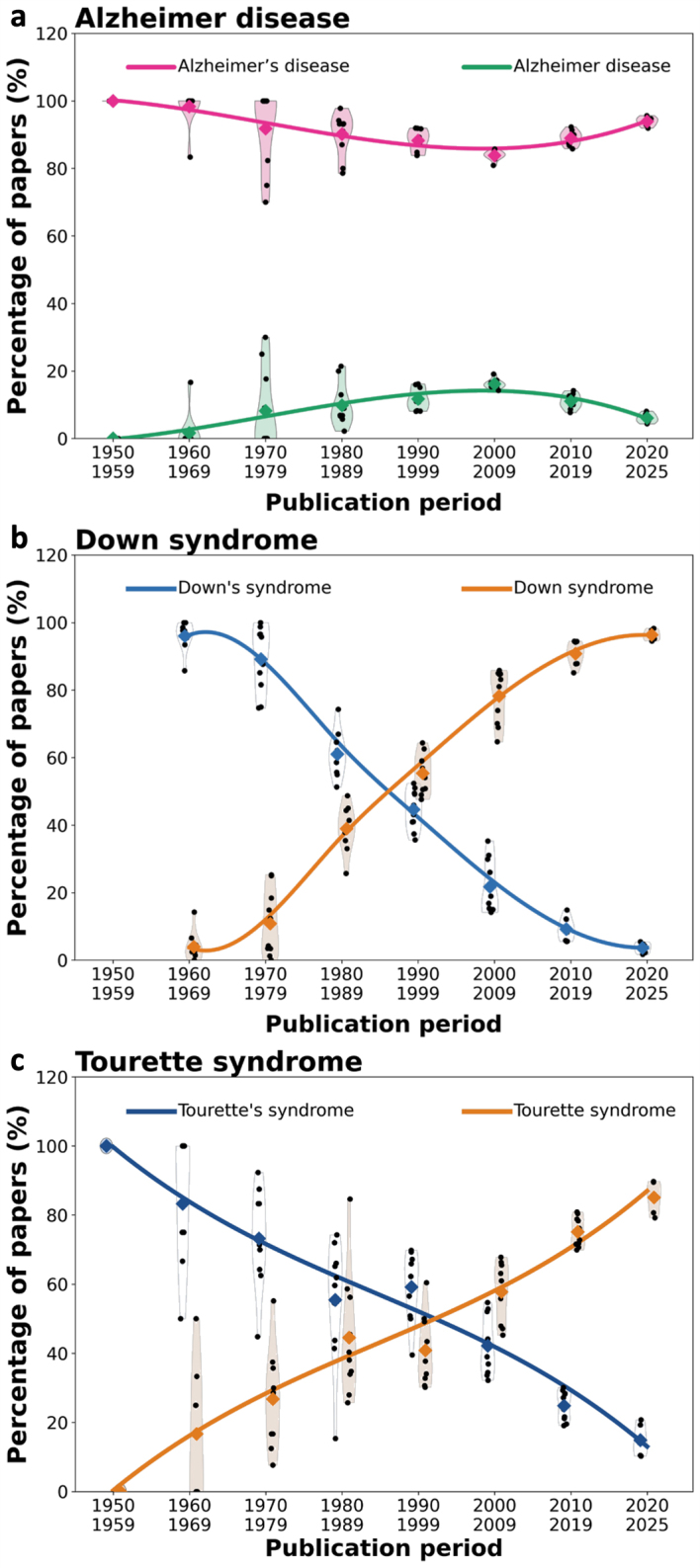
**Fig. 1**. Trajectories of possessive and non-possessive disease eponyms in the biomedical literature. **a.** Alzheimer disease. **b.** Down syndrome. **c.** Tourette syndrome. For each condition, the proportion of PubMed-indexed English-language article titles using the possessive versus the non-possessive eponym is shown by decade (1950–2025). Historical or biographic articles were excluded. Individual points represent yearly values; shaded violins illustrate the distribution within each decade; diamonds indicate decade means; solid curves show smoothed trajectories fitted to the decade means. Colors distinguish possessive and non-possessive forms. Data were generated using a Python script executed on the Google Colab platform. The figure illustrates the evolving balance between possessive and non-possessive disease eponyms across conditions over time.

To analyze whether recommendations favoring the non-possessive form of Alzheimer disease translate into changes in clinical and scientific usage, we quantitatively analyzed the trajectories of PubMed-indexed article titles from 1950 to 2025. As shown in **Fig. 1a**, the possessive form "Alzheimer's disease" overwhelmingly dominated the literature in the mid-twentieth century, accounting for nearly all titles in the 1950s and approximately 85–95 % of article titles today. The non-possessive form "Alzheimer disease" emerged gradually from the 1970s onwards but has never exceeded 10–15 % of titles. Thus, despite international and institutional recommendations favoring the non-possessive form, its adoption remains limited, and the possessive variant continues to predominate.

We then asked whether the persistence of the possessive eponym is unique to the Alzheimer field or more widespread in neurology and neuroscience. As an initial comparison, we analyzed Down syndrome [9], a condition of particular relevance because individuals with Down syndrome frequently develop cognitive decline, memory loss, and characteristic Alzheimer pathology due to increased APP gene dosage. Similar to the situation observed for the Alzheimer term, the possessive form "Down's syndrome" was used almost exclusively in the 1960s. In sharp contrast to the Alzheimer term, however, a continuous shift towards the non-possessive form occurred later on for Down syndrome (**Fig. 1b**).

Today, the non-possessive form "Down syndrome" is used almost exclusively. The shift from "Down's syndrome" to "Down syndrome" likely reflects the early influence of human genetics in this field. After Lejeune, Gautier, and Turpin identified trisomy 21 as the chromosomal cause of the condition in 1959 [10], the disorder acquired a precise biological definition that favored a descriptive rather than a possessive eponym.

As a second comparison, we analyzed Tourette syndrome [11]. Unlike Down syndrome, whose terminology was influenced by the identification of a defined genetic mechanism, Tourette syndrome remains primarily a clinically defined movement disorder, lacking a comparable mechanistic framework that might drive terminological change. According to our data, earlier literature predominantly used the possessive form "Tourette's syndrome", but since the 1990s, the non-possessive form "Tourette syndrome" has become predominant (**Fig. 1c**). Thus, even in the absence of a clear mechanistic definition, the neurology community has progressively adopted the non-possessive form.

The different trajectories observed for the possessive eponyms in Alzheimer disease, Down syndrome, and Tourette syndrome are striking and suggest that the continued use of “Alzheimer’s disease” reflects a field-specific resistance to terminological adaptation rather than a general reluctance within the neurology community to adopt modern nomenclature.

## Eponym heterogeneity in reviews and meta-analyses

Terminological heterogeneity has direct practical consequences for literature retrieval [12]. Systematic reviews and meta-analyses rely on the exhaustive identification of all relevant studies. Because two competing terms coexist for Alzheimer disease, searches restricted to a single term risk overlooking parts of the literature. The issue is therefore not merely grammatical but methodological.

**Figure 2:**
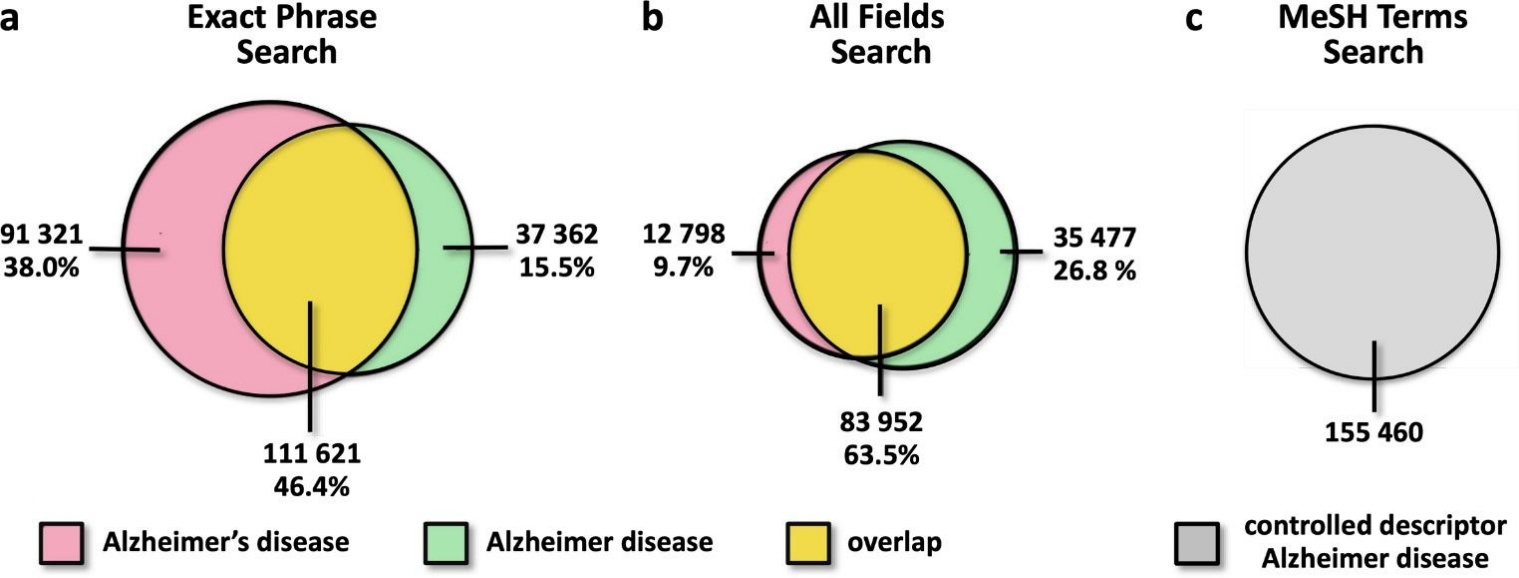
**Fig. 2**. Retrieval of PubMed references differs according to queries for possessive or non-possessive Alzheimer disease terms. a. Exact phrase searches for "Alzheimer's disease" and "Alzheimer disease" retrieve non-identical but partially overlapping sets of records. A large proportion of references are captured by only one search term. b. In all fields searches, the overlap between the two queries increases, but a substantial number of records is still missed by only one term, with the asymmetry now favoring the non-possessive form. Depending on the search type and term used (a–b), PubMed searches may thus miss up to 38 % of the literature, highlighting the substantial impact of terminological variation on literature retrieval. c. In contrast to free text searches, PubMed MeSH searches use the controlled descriptor Alzheimer Disease, i.e. the non-possessive form, yielding a uniform literature corpus. These analyses were performed across all article types, all languages, and all years since 1900, in contrast to the analyses presented in **Fig. 1**.

We thus examined whether PubMed searches using either "Alzheimer's disease" or "Alzheimer disease" retrieve identical, overlapping, or distinct sets of records. Our results show that the retrieved records are not identical. This discrepancy is observed both in exact phrase searches (**Fig. 2a**) and in all fields searches (**Fig. 2b**). Although the two record sets overlap substantially, each retrieves sizeable unique subsets of papers. With exact phrase searches, 38.0 % of records are retrieved only with "Alzheimer's disease" and 15.5 % only with "Alzheimer disease". In contrast, when all fields are searched, the asymmetry reverses: 26.8 % of records are retrieved only with "Alzheimer disease" and 9.7 % only with "Alzheimer's disease", reflecting the normalization of indexing terminology (**Fig. 2a–b**). Thus, depending on the search term used, up to one third of the literature may be excluded from retrieval. 

By contrast, PubMed searches based on MeSH (Medical Subject Headings) rely on the controlled descriptor "Alzheimer Disease" without the possessive 's. This controlled vocabulary normalizes terminology independently of the wording used in titles or abstracts, ensuring consistent retrieval across MeSH-indexed records (**Fig. 2c**).

These observations argue against the continued use of inconsistent terminology. The case for discouraging the term "Alzheimer's disease" is therefore not only stylistic but also epistemic; terminological inconsistency can fragment the evidence base and complicate comprehensive literature retrieval.

## Alzheimer eponyms, nosology, and disease mechanisms

Eponyms have long been useful in identifying disease entities and distinguishing them fromothers. Yet, they are often imprecise descriptors of biology. Alois Alzheimer originally described onesingle case. Since then, the field has expanded enormously, revealing marked heterogeneity inpathology, clinical presentation, and molecular mechanisms. Some cases show prominent synuclein co-pathology, others do not. Modifier genes, epigenetic factors, and differential vulnerability of neuronal systems all contribute to a growing spectrum of disease subsets.

As biomedical knowledge advances, the phrase "Alzheimer disease" itself increasingly functions less as a single sharply bounded entity than as a family of related pathological states. Parallel expressions such as "Alzheimer risk" further broaden the conceptual landscape. This explosion in mechanistic and nosological complexity highlights the limitations of inherited eponym terminology and supports the move toward more precise, standardized usage whenever possible.

## Conclusions

Why does the terminology shift from possessive to descriptive eponyms matter? First and foremost, it is a matter of accuracy and precision. After years of working as physician-scientists, we view Alzheimer disease as a biological and pathological entity; it is not something owned by any individual. Using the non-possessive form reminds us that this is a condition, not a possession.

Second, removing the possessive eponym from Alzheimer's promotes uniformity across different languages and cultures, which is crucial in our globalized world of medicine and science. Non-possessive eponyms are easier to translate and adapt internationally, helping healthcare professionals from different countries communicate more effectively [13]. In accordance with major medical authorities, including the World Health Organization, the non-possessive form is recommended.

Third, scientific language evolves, and with it the standards of clarity, precision, and professionalism. Historical convention explains why the possessive form remains common in parts of the Alzheimer field. Still, comparison with Down syndrome and Tourette syndrome shows that such conventions can change, and that neurology is fully capable of adopting the non-possessive form once consensus emerges.

So, the next time we write or speak about this devastating disorder, it is worth remembering that "Alzheimer disease" is not only the internationally recommended term but also the one that bestsupports clarity, precision, and comprehensive literature retrieval. Respect for history and respect for scientific rigour need not conflict. On the contrary, adopting evolving terminology standards is one way to honor both. 

## Disclosure statement

Generative artificial intelligence (AI) tools, including OpenAI’s GPT-5.3, were used to assist with code refinement, data verification, figure preparation, and limited text editing. No AI tools were used for data generation or interpretation. All content was independently reviewed and approved by the authors, who take full responsibility for the integrity and accuracy of the work.

## Conflict of interest statement

The authors declare no conflict of interest.
